# Supplementary material for: Fatal heart disease among cancer patients
Source: Nat Commun. 2020 Apr 24;11:2011. doi: 10.1038/s41467-020-15639-5 (PMC7181822; doi:10.1038/s41467-020-15639-5)
Supplement: Supplementary file 1 — Supplementary Information [file 41467_2020_15639_MOESM1_ESM.pdf]

COVER PAGE

Title: Fatal heart disease among cancer patients
Authors: Stoltzfus et al.

SUPPLEMENTARY INFORMATION

Supplementary Notes

Calendar period, registry, and diversity

The SEER program began in 1973 and is continually evolving [1]. With 36 years of longitudinal data, the database contains over 7 million cancer cases and has a comprehensive quality assurance process. Over time, data was collected from more areas of the US. Geographic areas were included based on two objectives: (1) the ability of a geographic cancer registry to maintain high-quality data (explained below), and (2) having a population that represents minority subpopulations.[1] The first areas included were Connecticut, Hawaii, Iowa, San Francisco/Oakland, and Detroit, followed by the metropolitan areas of Atlanta and Seattle/Puget Sound (SEER 9). The next iteration expanded upon the second objective, adding Los Angeles County and 4 counties from the San Jose/Monterey area (SEER 11). These counties included cases diagnosed after 1992. The next grouping additionally included 10 predominantly African American counties of rural Georgia and the Alaska Native American Tumor Registry (SEER 13). For cancers diagnoses after 2001, four additional areas were included: the remaining counties of California, Kentucky, Louisiana, and New Jersey (SEER 17). Funding from the Centers for Disease Control (CDC) is provided to these counties. Based on the inclusion of these areas, the SEER database is representative of the population of the USA, and this has been validated by external studies.[1] In the current analysis, the SEER 18 (adjusted for Hurricane Katrina Impacted Louisiana cases) and SEER 13 registries were used.

Early years of the SEER program includes fewer survivors than in later years due to the increased proportion of the US population that newer registries captures. Additionally, the proportion of death by index cancer is lower in later years. The rate of cancer in a population depends on a number of factors, including: number of patients living with this cancer from previous

years, number diagnosed within the calendar year, number dying during that year. These factors depend on cancer prevalence, incidence, screening trends, treatment methods and aggressiveness, coding variability, risk factors and comorbidities, and patient age. Certain cancers have an indolent course (e.g. prostate), and patients diagnosed in subsequent years are added to the cumulative count, increasing the number of prostate cancer patients relative to all others; for patients with aggressive cancers (e.g. pancreatic), the addition of patients diagnosed in subsequent years has little effect on the cumulative number because of high rates of mortality.

Age

SEER provides age-standard adult (age ≥15) cancer populations to calculate age-standardized survival, which is used to compare survival across time or different cancer populations with different age distributions. The standards provided are the International Cancer Survival Standard (ICSS) derived for three broad groups of cancer sites with similar patterns of incidence by age. ICSS 1 includes cancer sites with increasing incidence by age (most cancer sites; e.g. prostate). ICSS 2 includes cancer sites with broadly constant incidence by age (e.g. nasopharynx). ICSS 3 includes cancer sites that mainly affect young adults (e.g. testis). By using the appropriate standard, the age-standardized survival is theorized to be like the raw (un-weighted) survival. For each of the three ICSS populations, SEER*Stat provides weights by 5-year age bins using the age variable, Age recode with <1 year olds, and by five larger age groups, in the variable, Age Standard for Survival (15-44, 45-54, 55-64, 65-74, 75+), as described on the SEER website.

Quality assurance and completeness

SEER undergoes quality assurance using systematic, standardized, and periodic data collection procedure for all defined members of a defined cohort is performed to avoid surveillance

bias.[1] The case-finding audits are performed by a qualified member from each SEER registry under the direction of members of the National Cancer Institute. Auditors create an abstract that contains the primary site and the case finding source.[2] When performing audits, SEER adheres to two basic principles: auditing high quantity and high risk data. High quantity refers to disease sites that have the highest incidence and prevalence (e.g. breast, prostate, lung, colorectum); as well facilities that contribute the greatest percent of cases to the central database. Additionally, pathology laboratories are selected to review tissue from patients not seen at that hospital. High risk refers to cases that are likely to be miscoded (e.g. head and neck, hematopoietic diseases); compliance to new rules; and newly-reportable diseases.

Defining the cause of death

Mortality codes in SEER are assigned from death certificates, completed by the doctor caring for the patient at the time of demise. There is no single best method for calculating survival from cancer in the SEER program.[3] Different methods can give different outcomes, but for most variants considered the differences are small. For heart disease, there may be some discrepancy in the cause of death, since the death may because of the cancer itself, the cancer treatment, underlying heart disease, or a combination.

Cardiovascular-related deaths can be classified as six different overarching categories according to SEER. They include: diseases of the heart; hypertension without heart disease; cerebrovascular diseases; atherosclerosis; aortic aneurysm and dissection; other diseases of arteries, arterioles, capillaries. The patients included in the present study are coded as dying from diseases of the heart. A further breakdown of the ICD-9/-10 codes that fall into this category are provided in **Supplementary Table 1**.

Calculating Standardized Mortality Ratios

SMRs consist of two measures: (observed number of events, during time at risk) / (expected number of events in the reference population, during time at risk). SMRs may be calculated as a function of different times at risk, including time after diagnosis (i.e. the latency period) or age at diagnosis. When SMRs are calculated as a function of time after diagnosis, they provide the relative risk of death from one particular cause vs. the reference population. The reference population changes depending on the population and the time period. Thus, SMRs should not be compared to one another, and they would be expected to vary over different time periods or with different patient populations. Further, calculated SMRs may differ when using different SEER databases because (1) the observed number of events of interest among cancer patients may change, and (2) the number of events of interest in the reference population (i.e. the United States) also changes over the years.

Latency Exclusion Periods in Standardized Mortality Ratios

For SMRs calculated as a function of follow up time, SMRs during each window of time (e.g. at 1 year after diagnosis, 1-5 years after diagnosis, etc.) depend on the time at risk. With longer time at risk and more observed events, the confidence intervals become smaller, and measurements are more accurate. With a short time at risk (e.g. the first few months after diagnosis), or very few events (e.g. suicide), or among a niche patient cohort (e.g. Hodgkin lymphoma), the confidence intervals can widen dramatically.

In the first few months after diagnosis of cancer, patients often have an "introduction to the medical system;" i.e. a patient living in a rural area comes to a hospital where they are diagnosed with cancer, as well as many other comorbidities like heart disease, lung dysfunction, kidney failure, etc. The patient may die of any of these within a few months, but estimating the observed versus expected rate of death becomes difficult, and the confidence intervals for an SMR naturally

widen. Thus, some researchers, including our team, sometimes elect to exclude the first 2 months from the SMR calculations. While SMRs may actually be very high during this time, the confidence intervals are so wide that an accurate measure is not meaningful. Moreover, the absolute number of observed events in this time may be rather low, especially when the event of interest is rare. Thus, the overall SMRs for the entire follow up period (with or without the latency periods) tend to be relatively similar.

Data accessibility

The instructions to access the SEER data are provided below:

(1) Download the SEER*Stat software from the NCI website: https://seer.cancer.gov/seerstat/software/

(2) Open the program

(3) Click "File", "New,"

> **"MP-SIR"** Session to generate the SMRs. Note, this was used in Table 1, Figure 1, and Figure 3 of the current analysis.
>
> **"Case Listing"** to generate a list of patient cases diagnosed. Note, this was used in Table 1.
>
> **"Incidence"** to generate a list of the incidence of cancer or cause of death. Note, this was used in Table 2 and Figure 2 of the current analysis, and to generate the ORs.

(4) Click on the desired registry to use for each of the sessions. All the other data supporting the findings of this study are available within the article and Source Data file, and from the corresponding author upon reasonable request.

Supplementary Figure Legends

Supplementary Figure 1. Observed number of deaths due to primary cancer, diseases of the heart, non-index cancer, and other cause of death.

The y-axis depicts the observed number of deaths from each cause of death. The x-axis depicts the number of years since cancer diagnosis. Each graph represents a patient of the same primary disease site, as indicated by the title on the graph. The four colors represent the following causes of death: blue = primary cancer; orange = disease of the heart; gray = non-index cancer; yellow = other cause of death. Other cause of death includes any non-cancer related death that is recorded in the SEER database. For four of the six cancers presented, most patients die from their initial cancer than from any other cause as time since diagnosis increases. A large portion patients originally diagnosed with prostate cancer will die from another cause of death as time from initial diagnosis increases. Source data are provided as a Source Data file.

Supplementary Figure 2. Observed number of deaths due to primary cancer, diseases of the heart, non-index cancer, and other cause of death.

The y-axis depicts the observed number of deaths from each cause of death. The x-axis depicts the number of years since cancer diagnosis. Each graph represents a patient of the same primary disease site, as indicated by the title on the graph. The four colors represent the following causes of death: blue = primary cancer; orange = disease of the heart; gray = non-index cancer; yellow = other cause of death. Other cause of death includes any non-cancer related death that is recorded in the SEER database. For the six primary cancers presented, the majority of patients die from their initial cancer than from any other cause as time since diagnosis increases. Source data are provided as a Source Data file.

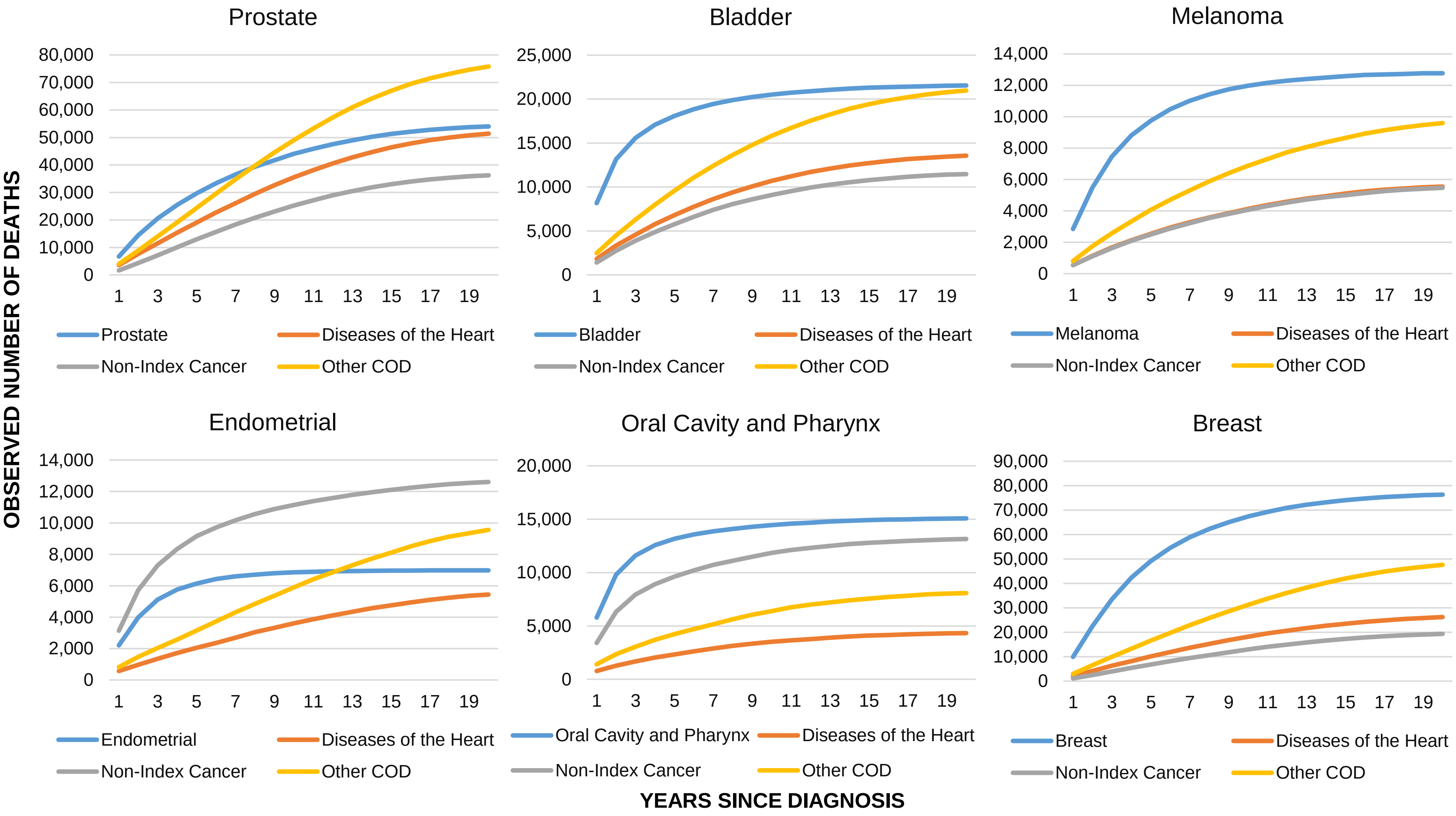

Supplementary Figure 1

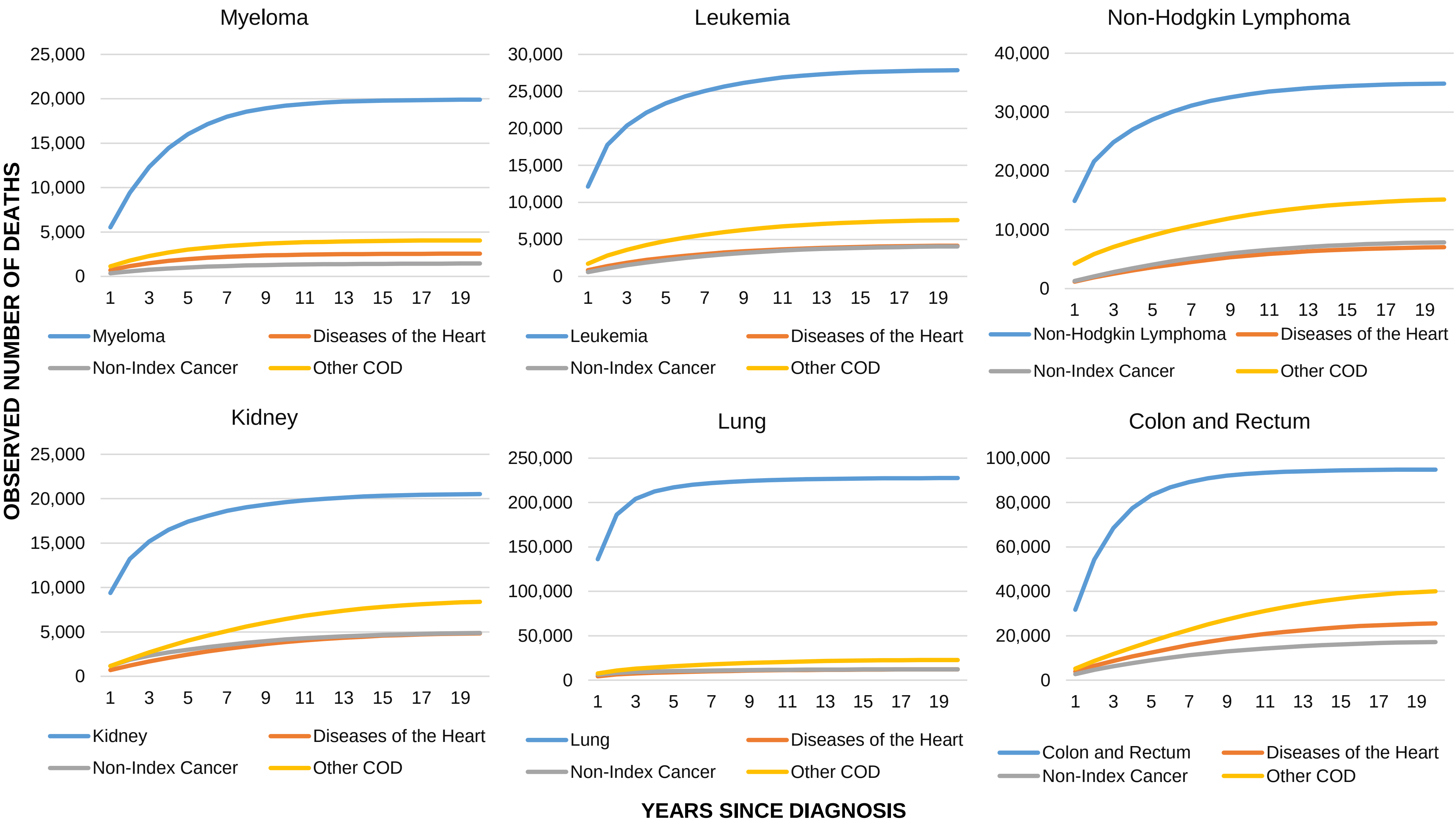

Supplementary Figure 2

Supplementary Table 1. ICD-9 and ICD-10 codes used for defining fatal heart disease.

ICD-9-CM diagnosis coding	Disease/condition	ICD-10-CM diagnosis coding	Disease/condition
390	Rheumatic fever without mention of heart involvement	I00	Acute rheumatic fever without heart involvement
391	Rheumatic fever with heart involvement	I01	Rheumatic fever with heart involvement
392	Rheumatic chorea	I02	Rheumatic chorea
393	Chronic rheumatic pericarditis	I05	Rheumatic mitral valve diseases
394	Diseases of mitral valve	I06	Rheumatic aortic valve diseases
395	Diseases of aortic valve	I07	Rheumatic tricuspid valve diseases
396	Diseases of mitral and aortic valves	I08	Multiple valve diseases
397	Diseases of other endocardial structures	I09	Other rheumatic heart diseases
398	Other rheumatic heart disease	I11	Hypertensive heart disease
402	Hypertensive heart disease	I13	Hypertensive heart and chronic kidney disease
404	Hypertensive heart and chronic kidney disease	I20	Angina pectoris
410	Acute myocardial infarction	I21	Acute myocardial infarction
411	Other acute and subacute forms of ischemic heart disease	I22	Subsequent ST elevation (STEMI) and non-ST elevation (NSTEMI) myocardial infarction
412	Old myocardial infarction	I23	Certain current complications following ST elevation (STEMI) and non-ST elevation (NSTEMI) myocardial infarction (within the 28 day period)
413	Angina pectoris	I24	Other acute ischemic heart diseases
414	Other forms of chronic ischemic heart disease	I25	Chronic ischemic heart disease
415	Acute pulmonary heart disease	I26	Pulmonary embolism
416	Chronic pulmonary heart disease	I27	Other pulmonary heart diseases
417	Other diseases of pulmonary circulation	I28	Other diseases of pulmonary vessels
420	Acute pericarditis	I30	Acute pericarditis
421	Acute and subacute endocarditis	I31	Other diseases of pericardium
422	Acute myocarditis	I32	Pericarditis in diseases classified elsewhere
423	Other diseases of pericardium	I33	Acute and subacute endocarditis
424	Other diseases of endocardium	I34	Nonrheumatic mitral valve disorders
425	Cardiomyopathy	I35	Nonrheumatic aortic valve disorders

426	Conduction disorders	I36	Nonrheumatic tricuspid valve disorders
427	Cardiac dysrhythmias	I37	Nonrheumatic pulmonary valve disorders
428	Heart failure	I38	Endocarditis, valve unspecified
429	Ill-defined descriptions and complications of heart disease	I39	Endocarditis and heart valve disorders in diseases classified elsewhere
		I40	Acute myocarditis
		I41	Myocarditis in diseases classified elsewhere
		I42	Cardiomyopathy
		I43	Cardiomyopathy in disease classified elsewhere
		I44	Atrioventricular and left bundle-branch block
		I45	Other conduction disorders
		I46	Cardiac arrest
		I47	Paroxysmal tachycardia
		I48	Atrial fibrillation and flutter
		I49	Other cardiac arrhythmias
		I50	Heart failure
		I51	Complications and ill-defined descriptions of heart disease

Supplementary Table 2. **Trend test for change in proportion of deaths from primary cancer vs. heart disease.**

Cancer Site	Chi-squared Value[1]	P-value
All Sites	123840	<0.001
Prostate	4383.3	<0.001
Breast	7136.6	<0.001
Colon and Rectum	22884	<0.001
Bladder	5943.9	<0.001
Melanoma	2203.6	<0.001
Lung	12373	<0.001
Kidney	3514.1	<0.001
Endometrial	4167	<0.001
Leukemia	1717.8	<0.001
Oral Cavity And Pharynx	2744.1	<0.001
Myeloma	30.9	<0.001
Non-Hodgkin Lymphoma	4299.9	<0.001

1- DF = 1; two-sided test

Supplementary References

1 Park, H. S., Lloyd, S., Decker, R. H., Wilson, L. D. & Yu, J. B. Overview of the Surveillance, Epidemiology, and End Results database: evolution, data variables, and quality assurance. *Current problems in cancer* **36**, 183-190, doi:10.1016/j.currproblcancer.2012.03.007 (2012).

2 *National Cancer Institute. Casefinding Studies - SEER Quality Improvement.*, <http://seer.cancer.gov/qi/tools/casefinding.html> (2016).

3 Boer, R. *et al.* (Statistical Research and Applications Branch, NCI, Bethesda, MD).
