# Supplementary material for: Fatal heart disease among cancer patients
Source: Nat Commun. 2020 Apr 24;11:2011. doi: 10.1038/s41467-020-15639-5 (PMC7181822; doi:10.1038/s41467-020-15639-5)
Supplement: Supplementary file 3 — Reporting Summary [file 41467_2020_15639_MOESM3_ESM.pdf]

nature research

Corresponding author(s): Nicholas G. Zaorsky

Last updated by author(s): Feb. 5, 2020

Reporting Summary

Nature Research wishes to improve the reproducibility of the work that we publish. This form provides structure for consistency and transparency in reporting. For further information on Nature Research policies, see Authors & Referees and the Editorial Policy Checklist .

Statistics

For all statistical analyses, confirm that the following items are present in the figure legend, table legend, main text, or Methods section.

n/a	Confirmed	
☐	☒	The exact sample size (*n*) for each experimental group/condition, given as a discrete number and unit of measurement
☐	☒	A statement on whether measurements were taken from distinct samples or whether the same sample was measured repeatedly
☐	☒	The statistical test(s) used AND whether they are one- or two-sided<br>*Only common tests should be described solely by name; describe more complex techniques in the Methods section.*
☐	☒	A description of all covariates tested
☐	☒	A description of any assumptions or corrections, such as tests of normality and adjustment for multiple comparisons
☐	☒	A full description of the statistical parameters including central tendency (e.g. means) or other basic estimates (e.g. regression coefficient) AND variation (e.g. standard deviation) or associated estimates of uncertainty (e.g. confidence intervals)
☐	☒	For null hypothesis testing, the test statistic (e.g. *F*, *t*, *r*) with confidence intervals, effect sizes, degrees of freedom and *P* value noted<br>*Give P values as exact values whenever suitable.*
☒	☐	For Bayesian analysis, information on the choice of priors and Markov chain Monte Carlo settings
☒	☐	For hierarchical and complex designs, identification of the appropriate level for tests and full reporting of outcomes
☒	☐	Estimates of effect sizes (e.g. Cohen's *d*, Pearson's *r*), indicating how they were calculated

Our web collection on statistics for biologists contains articles on many of the points above.

Software and code

Policy information about availability of computer code

Data collection	Cancer patients diagnosed between 1992 and 2015, were abstracted from the National Cancer Institute's Surveillance, Epidemiology, and End Results (SEER) program. SEER is a network of population-based incident tumor registries from geographically distinct regions in the US, covering 28% of the US population, including incidence, survival, and limited treatment information (e.g. radiation therapy, surgery, chemotherapy). The SEER registry includes data on sex, age at diagnosis, race, marital status, and year of diagnosis.
Data analysis	SEER*Stat 8.3.5, available through the National Cancer Institute, and Microsoft Excel 15.0.4 (Microsoft, Redmond, WA) were used for analysis.

For manuscripts utilizing custom algorithms or software that are central to the research but not yet described in published literature, software must be made available to editors/reviewers. We strongly encourage code deposition in a community repository (e.g. GitHub). See the Nature Research guidelines for submitting code & software for further information.

Data

Policy information about availability of data

All manuscripts must include a data availability statement. This statement should provide the following information, where applicable:

- Accession codes, unique identifiers, or web links for publicly available datasets
- A list of figures that have associated raw data
- A description of any restrictions on data availability

The source data supporting the findings of this study are provided as a Source Data file. Any additional information can be found within the supplementary information files or may be requested from the corresponding author. The data obtained for the current project from the SEER database are freely accessible to the public. The relevant session information, i.e. the user-submitted request, from in the current work and abbreviated data set (from SEER) are provided in Source Data file. We comply with all relevant ethical regulations. The datasets generated and analyzed during the current study are available in the SEER repository (https://seer.cancer.gov/seerstat/). The study was exempt from IRB review as these data are freely available via the National Cancer Institute SEER program. There are no participants in the study, and thus there is no consent form.

Field-specific reporting

Please select the one below that is the best fit for your research. If you are not sure, read the appropriate sections before making your selection.

- [ ] Life sciences
- [x] Behavioural & social sciences
- [ ] Ecological, evolutionary & environmental sciences

For a reference copy of the document with all sections, see nature.com/documents/nr-reporting-summary-flat.pdf

Behavioural & social sciences study design

All studies must disclose on these points even when the disclosure is negative.

Study description	The purpose of this study is to identify cancer patients at highest risk of fatal heart disease compared to (1) the general population and (2) other cancer patients at risk of death during the study time period. The study is a cross-sectional study involving only quantitative data. Patients with invasive cancer, as coded in the SEER database, were included in the analyses. We specifically looked at patients who died from heart disease.
Research sample	All patients with an invasive cancer diagnosis, diagnosed between 1992 and 2015, for whom data was available through the SEER database were included in the study analyses. Data were abstracted from the National Cancer Institute's Surveillance, Epidemiology, and End Results (SEER) program. SEER is a network of population-based incident tumor registries from geographically distinct regions in the US, covering 28% of the US population, including incidence, survival, and limited treatment information (e.g. radiation therapy, surgery, chemotherapy). For the current analysis, the SEER 18 and 13 registries were used. The SEER registry includes data on sex, age at diagnosis, race, marital status, and year of diagnosis. The data are freely accessible to the public. The study sample was selected by including all patients diagnosed with an invasive cancer, who were not diagnosed with cancer via autopsy or death certificate. This particular time period was selected because it provides us with a long enough follow-up period (10+ years) to compare how risk of heart disease changes with increased time since cancer diagnosis.
Sampling strategy	All patients coded as having invasive cancer in the SEER database were included in the analyses (a total of 7,529,481 cancer patients). Of these, 394,849 (5.24%) died of heart disease. These sample sizes were not specifically chosen, but rather included all data available in SEER at the time the data was pulled from the database.
Data collection	A case listing of patients with invasive cancer, diagnosed between 1992 and 2015, was abstracted from the SEER program. Variables pulled included patient ID, age, age at diagnosis, year of birth, month of diagnosis, year of diagnosis, race/ethnicity, sex, cancer site (including primary site), marital status, survival months, cause of death, and vital status. Incidence data and standardized mortality ratios (SMRs) for these patients were also collected from the SEER database. Data collection was done at one point in time by the corresponding author, Dr. Nicholas Zaorsky, from the SEER database. The data was copied from the SEER database into an Excel spreadsheet. The data was verified by authors Kelsey Stoltzfus and Ying Zhang.
Timing	Data for this study was obtained from the SEER database in December of 2018 and May of 2019.
Data exclusions	Patients diagnosed with cancer only through autopsy or death certificate were excluded (<1.5% of patients). Data were extracted for cancers with more than 10,000 person-years or more of survival time; thus, certain uncommon and aggressive cancers were excluded, including Kaposi's sarcoma, male breast cancers, and mesotheliomas. The cut-off point of 10,000 person-years of survival time was established pre-data analysis. Including these certain rare and aggressive cancers would not have allowed us to accurately depict the relationship between cancer and heart disease as these patients usually have a shorter survival time, decreasing the likelihood they'll die from heart disease.
Non-participation	Because the study design is using retrospective data, non-participation is not applicable.
Randomization	Because the study design is using retrospective data, randomization is not applicable.

Reporting for specific materials, systems and methods

We require information from authors about some types of materials, experimental systems and methods used in many studies. Here, indicate whether each material, system or method listed is relevant to your study. If you are not sure if a list item applies to your research, read the appropriate section before selecting a response.

Materials & experimental systems

n/a	Involved in the study
x	Antibodies
x	Eukaryotic cell lines
x	Palaeontology
x	Animals and other organisms
x	Human research participants
x	Clinical data

Methods

n/a	Involved in the study
x	ChIP-seq
x	Flow cytometry
x	MRI-based neuroimaging
